# Reversal of Platinum-based Chemotherapy Resistance in Ovarian Cancer by Naringin Through Modulation of The Gut Microbiota in a Humanized Nude Mouse Model

**DOI:** 10.7150/jca.96448

**Published:** 2024-06-11

**Authors:** Bingqing Xie, Xiaoni Zhou, Chuanlin Luo, Yilin Fang, Yufei Wang, Jing Wei, Liping Cai, Tingtao Chen

**Affiliations:** 1 Department of Obstetrics & Gynecology, The First Affiliated Hospital, Jiangxi Medical College, Nanchang University, Nanchang 330006, Jiangxi, China.; 2 National Engineering Research Center for Bioengineering Drugs and the Technologies, Institute of Translational Medicine, Jiangxi Medical College, Nanchang University, Nanchang 330031, Jiangxi, China.; 3 School of Pharmacy, Jiangxi Medical College, Nanchang University, Nanchang,330031, Jiangxi, China.; 4 Queen Mary School, Nanchang University, Nanchang 330031, China.

**Keywords:** ovarian cancer, cisplatin resistance, humanized model, inflammation, gut microbiota

## Abstract

As a chemotherapy agent, cisplatin (DDP) is often associated with drug resistance and gastrointestinal toxicity, factors that severely limit therapeutic efficacy in patients with ovarian cancer (OC). Naringin has been shown to increase sensitivity to cisplatin, but whether the intestinal microbiota is associated with this effect has not been reported so far. In this study, we applied a humanized mouse model for the first time to evaluate the reversal of cisplatin resistance by naringin, as well as naringin combined with the microbiota in ovarian cancer. The results showed that naringin combined with *Bifidobacterium animalis* subsp.* lactis* NCU-01 had an inhibitory effect on the tumor, significantly reducing tumor size (*p*<0.05), as well as the concentrations of serum tumor markers CA125 and HE4, increased the relative abundance of *Bifidobacterium* and *Bacteroides*, inhibit Toll-like receptor 4 (TLR4)/nuclear factor κB (NF-κB)-induced intestinal inflammation and increase the expression of intestinal permeability-associated proteins ZO-1 (*p*<0.001) and occludin (*p*<0.01). In conclusion, the above data demonstrate how naringin combined with *Bifidobacterium animalis* subsp. *lactis* NCU-01 reverses cisplatin resistance in ovarian cancer by modulating the intestinal microbiota, inhibiting the TLR4/NF-κB signaling pathway and modulating the p38MAPK signaling pathway.

## Introduction

Ovarian cancer (OC) is a prevalent malignancy in the female reproductive system with a significant mortality rate, positioning it as one of the most frequently encountered lethal tumors globally 1. At present, surgical procedures and chemotherapy are commonly employed as the primary approaches for addressing ovarian cancer. Cisplatin is a platinum-containing medication for OC treatment approved by the U.S. Food and Drug Administration 2, however, disease that recurs within 6 months of treatment with platinum-containing agents is considered platinum-resistant, and approximately 25% of patients suffer from cisplatin-resistant ovarian cancer 3,4. The discovery of novel medications that can delay cisplatin resistance is of the utmost importance, as the elevated recurrence rate due to cisplatin resistance imposes a significant burden on both ovarian cancer patients and society.

Recent research has indicated that incorporating citrus fruits into the human diet may have a preventive effect on cancer and hinder its growth 5-7. There is proof that naringin obstructs the proliferation of tumor cells and triggers apoptosis in breast, bladder, and cervical cancer cells 8. Additionally, this compound possesses a broad spectrum of pharmacological properties, encompassing antioxidant, anti-inflammatory and anticancer activities 9. Moreover, the cancer-inhibiting attributes of naringin are linked to the modulation of various signaling pathways, including nuclear factor E2 related factor 2 (Nrf2), nuclear factor kappa-B (NF-κB), Phosphatidylinositol-3-kinase/Automatischer Kassentresor/Mammalian Target of Rapamycin (PI3K/Akt/mTOR), c-Jun N-terminal kinase (JNK), extracellular regulated protein kinases (ERK) and p38 Mitogen-activated protein kinase (p38 MAPK) 10. At present, limited research has been conducted on the sensitizing impact of naringin in adjuvant chemotherapy for ovarian cancer. Further investigation is needed to determine if it can enhance the effectiveness of chemotherapeutic agents and to understand its mechanism of action.

Multiple research studies have indicated that the microbiota in the digestive system has a significant impact on conditions such as inflammatory bowel disease, diabetes, Parkinson's disease and cancer 11-12. The gut microbiota (GM) is a symbiotic community consisting of countless bacteria that have a significant impact on human well-being 13. Toxic gastrointestinal reactions may occur as a result of imbalances in the gut microbiota during cancer radiotherapy. Hence, research conducted on both humans and animals has demonstrated that the gut microbiota can regulate immune responses against cancer and reduce the harmful side effects linked to cancer treatment 14-16. On the other hand, intestinal microbiota imbalance is characterized by a decrease in the number of beneficial microorganisms such as *bifidobacterial* 17. This leads to a decrease in the thickness of the intestinal mucosa, an increase in permeability and a weakening of the intestinal barrier. As a result, pro-inflammatory cytokines such as tumor necrosis factor-alpha (TNF-α) are stimulated, initiating pro-inflammatory mechanisms that could potentially aid in the advancement and growth of cancer 18. Some studies have shown that *Bifidobacterium animalis* helps maintain a healthy intestinal microbiota, improves intestinal health, protects against diarrhea, and helps mitigate the possible adverse effects of antibiotic treatment 19. Therefore, modulating the composition of the gut microbiota could be used as a way to improve the effectiveness of tumor treatments and related drugs.

At present, two categories of mouse models are utilized for ovarian cancer: wildtype mice injected with cells of murine origin and mice with compromised immune systems injected with cells of human origin. The distinction lies in the fact that the former preserves the unaltered immune system, whereas the latter replicates the invasive mechanism of human cancer cells. These two models are inadequate to examine the impacts of radiotherapy and colony interactions on tumors, thus necessitating the discovery of an alternative model that can more accurately replicate the immune microenvironment of humans. By transplanting human immune cells into mice with severe immunodeficiency, a mouse model with a functionally human immune system is created, which closely resembles the human immune system 20. Thus, it can be used for antitumor efficacy and pharmacodynamic studies using human cancer cells in mice containing immune components of humans 21.

The aim of our study was to evaluate naringin's ability to reverse platinum-based chemotherapy resistance in ovarian cancer by modulating the gut microbiota. To this end, we constructed a humanized mouse model to evaluate the therapeutic efficacy of naringin and *Bifidobacterium animalis* subsp. *lactis* NCU-01, combined with chemotherapeutic agents, on ovarian cancer and its mechanism of action by analysing the tumor volume and determining serum tumor markers, histopathological alterations and changes in the intestinal microbiota. Our findings may provide data support for future clinical applications of naringin and NCU-01.

## Materials and Methods

### *In vitro* experiments

#### Cell culture

A2780 and A2780/DDP cell lines were provided by the research team led by Prof. Tan of the Second Clinical Medical College of Nanchang University and cultured in RPMI-1640 medium supplemented with 10% fetal bovine serum, 100 U/mL penicillin and 100 U/mL streptomycin. Experiments were performed on cells in the exponential growth phase at an incubator temperature of 37°C, 5% CO_2_, and passaged every 2-3 days. The remaining cells were stored in liquid nitrogen.

#### MTT test

The MTT assay was used to evaluate the optimal concentration of naringin, the cisplatin resistance rate and the effect of naringin combined with cisplatin on human ovarian cancer cells. A2780/DDP cells in the logarithmic growth phase were removed, and 2 × 10^4^ cells/well were inoculated into 96-well plates and incubated overnight in an incubator to ensure cell attachment. Using an electronic balance, 5.8 mg of naringin powder (Solarbio, China) was weighed out, 1 mL of DMSO was added, blown and mixed, and then decontaminated using a disposable filter tip to obtain the original liquid at a concentration of 10 mmol/L. Subsequently, naringin was applied to the resistant cells at various concentrations (5, 10, 20, 40 and 80 µmol/L), and after 48 hours of incubation, the photometric (OD) values at 490 nm were measured and averaged to obtain a cytotoxic-free dose of naringin. Then, different concentrations of cisplatin (1.25, 2.5, 5, 10 and 20 µg/mL) were applied to A2780 and A2780/DDP cells in logarithmic growth phase, and the cell growth rate and 50% inhibitory concentration (IC_50_) were calculated. Finally, A2780/DDP cells were exposed to 20 µmol/L naringin in combination with different concentrations of cisplatin (1.25, 2.5, 5, 10 and 20 µg/mL), the OD values at 490 nm were measured after 48 hours of co-incubation and the average values were taken to calculate the multiplicity of resistance reversal.

#### Western blotting

Cells of the A2780/DDP cell line were collected during the logarithmic growth phase and divided into four different groups, Group M: A2780/DDP cells; Group MN: A2780/DDP cells + Naringin (20 µmol/L); Group MD: A2780/DDP cells + DDP (10 µg/mL); Group MND: A2780/DDP cells + DDP (10 µg/mL) + Naringin (20 µmol/L), and incubated for 24 hours. To prepare protein samples, cells were lysed using a protease-based RIPA lysis solution on ice for a duration of 30 minutes, and then centrifuged. Following polyacrylamide gel electrophoresis of proteins using a 10% SDS-PAGE gel, the proteins were transferred to a PVDF membrane, and nonspecific binding sites were blocked with a 5% skimmed milk solution for 90 minutes at room temperature. The membrane was then incubated overnight with a specific primary antibody (Supplementary materials Table SⅠ) and subsequently with a secondary antibody for 1 hour. Finally, the image was developed by adding ECL Light Emitting Reagent, and the grey-scale value of the protein bands was calculated using Image J, with β-actin serving as the internal reference.

#### Construction of a humanized nude mouse model

##### Separation of T lymphocytes from human peripheral blood

Peripheral blood was collected from healthy individuals in a sterile manner and treated with heparin as an anticoagulant for separation of peripheral blood mononuclear cells (PBMCs) by Ficoll density gradient centrifugation. Subsequently, the anticoagulated blood was supplemented with human peripheral blood lymphocyte isolation solution (Beijing Solebo Technology Co., Ltd.), and separated into three layers by centrifugation. At the interface of the upper and middle layers, a narrow band of white cloudy material, predominantly composed of single nucleated cells known as PBMCs was observed. A thin tube was inserted into the hazy section, and the PBMCs were extracted and transferred to a different 15 mL tube. Cells were washed with PBS, centrifuged to discard the supernatant, resuspended in PBS and centrifuged again. This process was repeated twice. Following the final centrifugation, the liquid above the sediment was removed, and the cells were reconstituted in RPMI-1640 solution with 10% foetal bovine serum. This mixture was then carefully injected into a nylon wool column and placed in a water bath. Human peripheral blood T-lymphocytes (HuPBTL) were discarded after washing the column with complete medium.

##### Animals and immune system construction

Eighty inbred female BALB/c nude mice aged 4-5 weeks and weighing 15-18 g were provided by Changzhou Cavins Laboratory Animal Co. Ltd (Jiangsu, China) and housed in an SPF environment with a constant temperature of 26-28°C and humidity maintained at 55% and were given food and water ad libitum. After one week of adaptive feeding, the cell density of isolated human T lymphocytes was adjusted to 8 × 10^6^/ml, and 8 × 10^6^ HuPBTL (200 μL) were injected via tail vein to establish the HuPBTL-nude mouse model. The reaction, presence or absence of rash, feeding, drinking, mental status and death of humanized nude mice were observed daily.

##### Flow cytometry

The tails of mice were clipped to collect 100-200 µL of peripheral blood in anticoagulation tubes, and murine anti-human CD3-FITC monoclonal antibody (4A BIOTECH, China) was added, and incubated for 20 min away from light. Erythrocytes were fully lysed for 10 min at room temperature, and 2 mL of PBS solution was added and mixed well. The sample was centrifuged and the supernatant discarded, 500µL of PBS solution was added, and the results were analysed by using a Beckman DxFLEX flow cytometer (Beckman, USA).

### *In vivo* experimental study of the effect of naringin on OC treatment

#### Experimental design

To investigate the impact of naringin on the progression of OC, a total of 40 humanized nude mice were successfully created and randomly divided into five groups. A2780/DDP cells were cultured and adjusted to a cell density of 2 × 10^6^ cells/mL. Subsequently, A2780/DDP cells were subcutaneously injected into the right dorsal lumbar region of 32 humanized nude mice using a 1mL syringe, and tumor growth was monitored with the following treatments: (1) Group C: 200 μL saline injected intraperitoneally for 10 days, n=8; (2) Group M: an OC model was established as described above and 200 μL of saline was injected intraperitoneally for 10 days, n=8; (3) Group MN: OC model with continuous administration of 200 μL naringin (2.0 mg/kg/d), by injection for 10 days, n=8; (4) MD group: OC model with continuous administration of 200 μL cisplatin (2.5 mg/kg/d), injected intraperitoneally for 10 days, n=8; (5) MND group: OC model with continuous administration of 200 μL cisplatin (2.5 mg/kg/d) and 200 μL naringin (2.0 mg/kg/d), injected intraperitoneally for 10 days, n=8. In order to reduce any potential bias, all animal experiments were conducted from 9:00 a.m. to 12:00 a.m. Throughout the duration of the experiment, every animal successfully completed the study. Peripheral blood was collected by clipping the tails of the rats again before administration to test the constructed immune system.

#### Sample collection

The body weight and graft tumor size of each mouse were measured the day after drug withdrawal, blood samples were collected from the mice before euthanasia and serum was obtained by centrifugation at 3500 rpm (R=13.5 cm) for 20 min and stored at -80°C together with faecal samples. The mice were then anesthetized with isoflurane gas and then euthanized 22. Two millilitres of isoflurane were aspirated onto a skimmed cotton ball and placed in the lower layer of a desiccator, and the mice were placed in the desiccator until the respiratory rate dropped to a lower level and then euthanized. Tumor and colon tissues of mice were collected for tumor size was measurement, and the volume was expressed by the formula of length × width × width × 0.5. A portion of the tumor tissue was fixed in 4% paraformaldehyde buffer for HE staining and immunohistochemical analysis, and the rest was stored at -80°C for future molecular biology experiments, such as protein blotting.

#### ELISA

The levels of tumor markers specific to ovarian cancer in serum samples were assessed by measuring the levels of CA125 and HE4 in mouse serum. This was performed using the suitable CA125 ELISA kit (from Wuhan, China) and HE4 ELISA kit (from Abcam). The absorbance (OD value) was measured using an enzyme meter and the concentration was calculated.

#### H&E and immunohistochemistry

Tumor tissues from every group underwent fixation, dehydration, and embedding using 4% paraformaldehyde. Subsequently, they were fixed, embedded in paraffin and sliced into tissue sections measuring 5 µm in thickness. After hydration with xylene and ethanol for approximately 5-6 minutes, the sections underwent three water washes, followed by staining with haematoxylin-eosin (H&E) and examination under a microscope.

Immunohistochemistry was conducted on paraffin-embedded tumor specimens using an anti-Ki67 antibody (1:10 000, GB11499, Servicebio, China). Following rinsing in PBS, a 50-minute incubation with HRP secondary antibody (1:200, G23303, Servicebio, China) was conducted. The slides were then dehydrated, sealed and subjected to colour development detection by adding 3,3'-diaminobenzidine. Finally, images were captured using a light microscope.

#### Western blotting

The tumor tissues were weighed and then placed on ice and then lysed using RIPA lysis solution (Solarbio, China, R0010) with the addition of 1 mM phenylmethylsulfonyl fluoride (PMSF). The lysates were homogenized and centrifuged in a centrifuge to obtain protein samples. Using polyacrylamide gel electrophoresis with 8-10% SDS-PAGE gels, proteins were separated, electrotransferred onto PVDF membranes and then sealed with a 5% skimmed milk solution for 90 minutes at room temperature. Subsequently, the membrane was incubated overnight with a specific primary antibody (Supplementary materials Table SⅡ), washed three times and incubated with the appropriate secondary antibody at room temperature for 1 hour. The expression of these specific proteins was analysed by exposing and imaging them using a gel imaging system equipped with chemiluminescent solutions (Thermo Fisher, 32209).

#### 16S rRNA high-throughput sequencing

Following the guidelines provided by the manufacturer, genomic DNA was isolated from mouse faeces using a genomic DNA kit (OMEGA Bio-Tek, Norcross, GA, USA, M5635-02). Utilizing universal primers for PCR amplification of the V4 region of the bacterial 16S rRNA gene, the resulting PCR products were subjected to sequencing on the Illumina NovaSeq platform. Finally, the QIIME2 software (version 3.2.0) was utilized for initial analysis of the sequencing data. This analysis aimed to assess α- and β- diversity. The software was also employed to detect variations in taxa abundance, conduct cluster analysis and perform partial least squares discriminant analysis.

### *In vivo* experiments confirm the effect of the microbiota on OC

#### Bifidobacterium animalis culture

A strain of *Bifidobacterium animalis* subsp. *lactis* NCU-01 (Laboratory of Translational Medicine, Nanchang University) was previously identified and isolated from the faeces of centenarians. Botulinum Selective Medium (BSM) was prepared by adding 100mL distilled water to 6.33g MRS Powder and 0.2% cysteine. *Bifidobacterium animalis* was cultured in BSM medium.

#### Experimental design

To confirm the role of the microbiota in OC progression, a total of 40 humanized nude mice were randomly divided into five groups. A2780/DDP cells, with a density of 2 × 10^6^ cells/mL, were incubated and then subcutaneously injected into 32 humanized nude mice on the right dorsal lumbar side using a 1 mL syringe to examine tumor growth and the following treatments were performed. (1) Group C: 200 μL of sterile saline by gavage for 10 days, n=8; (2) Group M: an OC model was established as described above, and 200 μL of sterile saline was administered by gavage for 10 days, n=8; (3) Group MND: In an OC model, 200 μL of cisplatin (2.5 mg/kg/d) and 200 μL of naringin (2.0 mg/kg/d) were injected intraperitoneally for 10 days, n=8; (4) MP group: In an OC model, 10^9^ colony forming units (CFU) of *Bifidobacterium animalis* subsp. *lactis* NCU-01were administered by continuous gavage for 10 days, n=8; (5) MNDP group: In an OC model, 200 μL of cisplatin (2.5 mg/kg/d) and 200 μL of naringin (2.0 mg/kg/d) were injected intraperitoneally, and 10^9^ colony forming units (CFU) of *Bifidobacterium animalis* subsp. *lactis* NCU-01 were administered by gavage, for 10 days, n=8. To minimize the systematic error, all animal experiments were conducted between 9:00 and 12:00, and all animals survived during the experiments. Peripheral blood was collected by clipping the tails of the rats again before drug administration to test the constructed immune system.

#### Sample collection

Following discontinuation of the drug, samples of faeces and serum were obtained prior to euthanasia. The serum samples were then centrifuged, and subsequently stored at a temperature of -80°C along with the faeces. After isoflurane gas administration for anaesthetic purposes and euthanasia 22, the tumor and colon tissues were gathered. The tumors were measured to determine their size, and their volume was calculated using the formula length × width × width × 0.5. A section of the tumor and colon tissues fixed in 4% paraformaldehyde buffer underwent HE staining and immunohistochemistry, while the remaining tissues stored at -80°C were subjected to q-PCR and western blotting analysis.

#### H&E and immunohistochemistry

Samples from tumors and colons were preserved in a 4% paraformaldehyde solution, rehydrated and then encased in paraffin wax. The tissues were sliced lengthwise and sequentially into 5μm sections, stained with haematoxylin and eosin, mounted onto slides, and observed under a light microscope for morphological analysis.

The formalin-fixed, paraffin-embedded tumor specimens were subjected to immunohistochemistry using the anti-Ki67 antibody (1:10 000, GB11499, Servicebio, China). Following PBS rinsing, the sections were treated with HRP secondary antibody (1:200, G23303, Servicebio, China) for a duration of 50 minutes. Subsequently, the slides were dehydrated, sealed, and subjected to colour development detection by adding 3,3'-diaminobenzidine. An Olympus I-71 (Olympus, Japan) was used for image acquisition.

#### ELISA

In order to assess the levels of tumor markers and inflammatory factors specific to ovarian cancer in serum samples, the expression of CA125 and HE4 in mouse serum was measured using ELISA kits for CA125 (Wuhan, China) and HE4 (Abcam). To determine the expression levels, the serum of mice was analysed using the corresponding kits to measure the levels of IL-6, IL-1β, and TNF-α. To calculate the concentration, the absorption (OD value) at 450 nm measured using an enzyme marker.

#### Western blotting

The tumor and colon tissues were weighed, and then lysed using RIPA lysis solution (Solarbio, China, R0010) with the addition of 1 mM phenylmethylsulfonyl fluoride (PMSF) on ice to prevent protein hydrolysis. The lysed samples were homogenized and centrifuged to obtain protein samples. These protein samples were separated using 8-10% SDS-PAGE. Following the electrothermal separation of proteins, PVDF membranes were blocked with a 5% skimmed milk solution for 90 minutes. After that, the membranes were incubated overnight at 4°C with specific primary antibodies (Table SⅠ of the Supplementary Material). After three washes, the sample was incubated with the secondary antibody for one hour. Ultimately, the analysis of these particular proteins was conducted by exposure to a chemiluminescent solution and imaging using a gel imaging system (Thermo Fisher, 32209).

#### DNA preparation and RT-qPCR

To assess the levels of pertinent microorganisms in mouse faeces, DNA was extracted from the faeces using a Faecal DNA Isolation Kit following the instructions provided by the manufacturer. Subsequently, RT-qPCR was conducted using a 2 T5 Fast qPCR Mix (SYBR Green I) kit, with a total of 40 cycles consisting of 30 seconds of pre-purification at 95°C, 5 seconds of denaturation at 95°C and 30 seconds of annealing at 60°C. The reaction was heated to 95°C for 5 seconds and cooled to 60°C for 30 seconds. By employing the 2^-ΔΔCq^ technique, we examined bacterial expression in faecal samples and assessed the levels of microbial expression (Primer sequence is shown in supplemental materials Table SⅡ).

### Statistical analysis

The analysis and plotting were performed using Prism version 9.0 software, and all values are presented as the mean ± standard deviation (SD). Statistical significance was determined using Tukey's multiple comparison test and one-way analysis of variance, and significant results are indicated by *p*<0.05 (*p<0.05, **p<0.01, ***p<0.001).

## Results

### Naringin inhibits tumor cell proliferation and reverses resistance to cisplatin (DDP)

**Figure 1:**
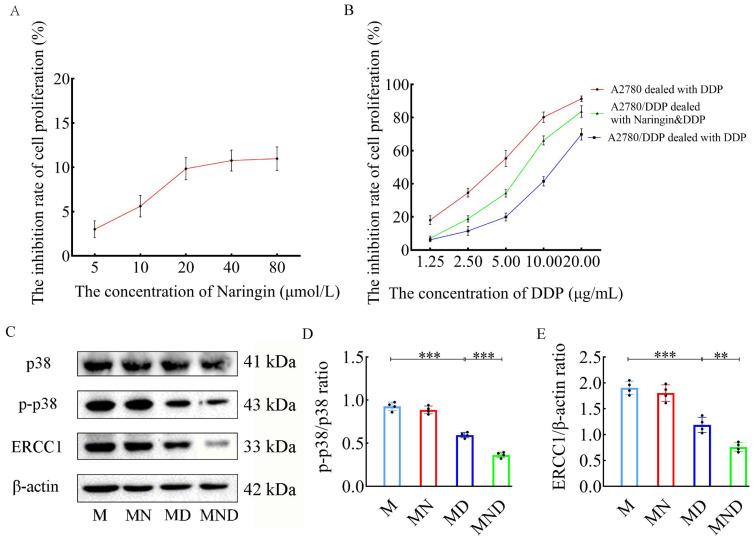
Reverse effect of naringin on cisplatin resistance in ovarian cancer cells. (A) The inhibition rate of cell proliferation in different concentrations of naringin. (B) The inhibition rate of cell proliferation in different concentrations of DDP. (C) Effect of different drugs on the protein expression in tumour cell (n = 4). (D,E) Relative expression of p-p38/p38 and ERCC1 in tumour cells by ImageJ software (n = 4). Data are presented as means ± SD. Student t test for two groups comparison. *p< 0.05, **p< 0.01, ***p< 0.001. M stands for model control group, MN stands for model + naringin group, MD stands for model + DDP group, MND stands for model+ naringin+ DDP group.

To assess the inhibitory impact on the proliferation of tumor cells and the reversal of DDP resistance using the optimal concentration of naringin, relevant experiments were performed on A2780/DDP cells. According to the findings of the MTT assay, there was a notable disparity in naringin concentrations exceeding 20 µmol/L after 48 hours (Figure 1A). Consequently, subsequent experiments were conducted using the noncytotoxic dose of 20 µmol/L naringin at the optimal time. After the tumor cells were subjected to various concentrations of cisplatin for 48 hours, the IC_50_ for A2780 cells was 3.931, whereas for A2780/DDP cells, it was 12.022. The inhibition of A2780 cells was greater than that of A2780/DDP cells when exposed to the same incubation time and cisplatin concentration, indicating a more pronounced resistance of A2780/DDP cells to cisplatin. Subsequently, we employed 20 µmol/L naringin in conjunction with varying amounts of DDP to co-culture A2780/DDP cells for a duration of 48 hours. Remarkably, we observed a dose-dependent suppression of cellular growth, with an IC_50_ value of 12.022 for A2780/DDP cells when DDP was administered as a standalone treatment. Furthermore, the IC_50_ value decreased to 6.818, when naringin and DDP were combined (Figure 1B). Hence, the multiplicity of cisplatin resistance was 3.058 and the reversal multiplicity of naringin was 1.763, suggesting that naringin possessed the ability to counteract cisplatin resistance.

To gain a deeper understanding of naringin's mechanism, we employed western blotting to analyse the protein expression related to the p38MAPK signaling pathway, specifically p38, phosphorylated p38 and ERCC1, in tumor cells (Figure 1C). In the MD group, the p-p38/p38 ratio (0.05945±0.032 vs 0.926±0.049, *p*<0.001), and the ERCC1 protein level (1.187±0.149 vs 1.905±0.126, *p*<0.001) showed a significant decrease compared to the M group (Figure 1D-E). No notable disparity was observed in the ratio of p-p38 to p38 and the level of ERCC1 protein between the MN and M groups. In the meantime, the MND group exhibited a significantly lower overall p-p38/p38 ratio (0.365±0.031 vs 0.05945±0.032, *p*<0.001) and ERCC1 level (0.760±0.088 vs 1.187±0.149, *p*<0.01) compared to the MD group. Additionally, the p38 protein level remained consistent across all four groups. The findings indicate that naringin has the potential to counteract cisplatin resistance, leading to an enhancement of the anticancer impact.

### Naringin combined with DDP synergistically overcomes drug resistance and enhances anti-tumor effects in a humanized ovarian cancer model

**Figure 2:**
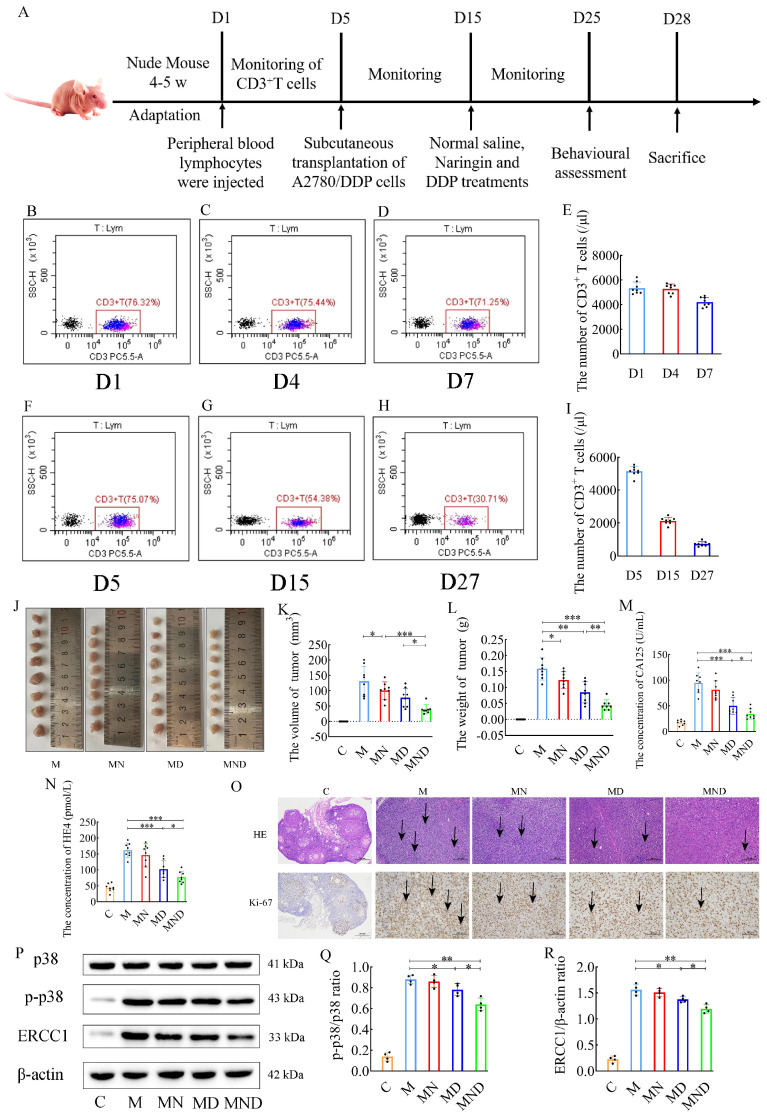
Construction of human immune system and reverse effect of naringin on cisplatin resistance in mice. (A) Schematic of animal experiments: Group C: 200 μL saline injected intraperitoneally for 10 days; Group M: OC model was intraperitoneally injected with 200 μL normal saline for 10 days; Group MN: OC model was intraperitoneally injected with 200 μL naringin (2.0 mg/kg/d) for 10 days; Group MD: OC model was intraperitoneally injected with 200 μL cisplatin (2.5 mg/kg/d) for 10 days; Group MND: OC model was intraperitoneally injected with 200 μL cisplatin (2.5 mg/kg/d) and 200 μL naringin (2.0 mg/kg/d) for 10 days. (B-I) T cells detected by flow cytometry in humanized mice. (J-L) Weights and volumes of tumours in different treatment groups (n = 8). (M-N) CA125 and HE4 level in serum (n = 8). (O) H&E staining images of tumour tissues were presented (scale bar = 200 μm) and Immunohistochemistry of Ki67 expression in tumour tissues (scale bar = 100 μm). (P-R) Relative expression of p-p38/p38 and ERCC1 in tumour tissues by ImageJ software (n = 4). Data are presented as means ± SD. Student t test for two groups comparison. *p< 0.05, **p< 0.01, ***p< 0.001. C stands for blank model group, M stands for model control group, MN stands for model + naringin group, MD stands for model + DDP group, MND stands for model+ naringin+ DDP group.

Naringin is being investigated for its effects on drug-resistant ovarian cancer; we chose a humanized nude mouse ovarian cancer model to better simulate the immune environment of the human body, and the entire experiment is shown in Figure 2A. Peripheral blood testing of humanized nude mice detected human CD3^+^ T cells in all of them (Figure 2B-E). There was no obvious weight change in the nude mice in any group, and there were no adverse reactions such as skin rash or diarrhoea. Flow cytometry was performed continuously on the peripheral blood of the mice during the experimental process, and the presence of human-derived CD3^+^ T cells was detected in subcutaneous tumor implantation, drug intervention and prior to euthanasia (Figure 2F-I), confirming that immunity could persist in the nude mice throughout the experimental process.

Additionally, in comparison to the M group, both the utilization of DDP alone and the combination of naringin and DDP resulted in a significant decrease in tumor volume (131.384±48.564 in the M group, 77.625±30.793 in the MD group, and 39.688±15.129 in the MND group) (Figure 2J-L). Furthermore, serum tumor markers showed that group M had significantly higher levels of CA125 and HE4 than group C. Serum CA125 (50.248±16.536 vs. 94.638±19.657, *p*<0.001) and HE4 (101.998±26.598 vs. 161.763±22.521, *p*<0.001) in group MD levels were significantly lower than those in group M, while treatment with naringin alone had no significant effect on serum CA125 and HE4 levels (*p*>0.05) (Figure 2M-N). By H&E staining and immunohistochemical analysis, we observed that the tumor cells in the model group (group M), the drug intervention group (group MN, group MD), and the combined treatment group (group MND) exhibited significant morphological heterogeneity and structural disorders compared with the control group (group C). Strikingly, the tumor cells in the MND group after the combined treatment were arranged in a similar manner to the control group, and the proliferative activity was effectively inhibited as reflected by the significant decrease in the expression level of Ki67 (Figure 2O). Through western blotting, we assessed the relevant proteins in the p38 MAPK signaling pathway in the tumor tissues and compared them to those in the DDP chemotherapy group alone. Interestingly, the combination of naringin and DDP led to a significant decrease in the levels of p-p38/p38 ratio (*p*<0.05) and the ERCC1 (*p*<0.05) proteins level. However, there was no significant difference in the p38 protein level between the groups (Figure 2P-R). The above data suggest that naringenin is able to reverse cisplatin resistance in ovarian cancer cells by decreasing the expression of ERCC1 in the cells, and its mechanism of action involves the p38MAPK signaling pathway.

### Modulation of intestinal microbial community by DDP and naringin

The gut microbiota, which is increasingly associated with various diseases such as cancer, plays a crucial role in maintaining human health 23-26. In this study, our aim was to validate the impact of chemotherapy on the gut microbiota in mice that underwent ovarian cancer xenografts.

**Figure 3:**
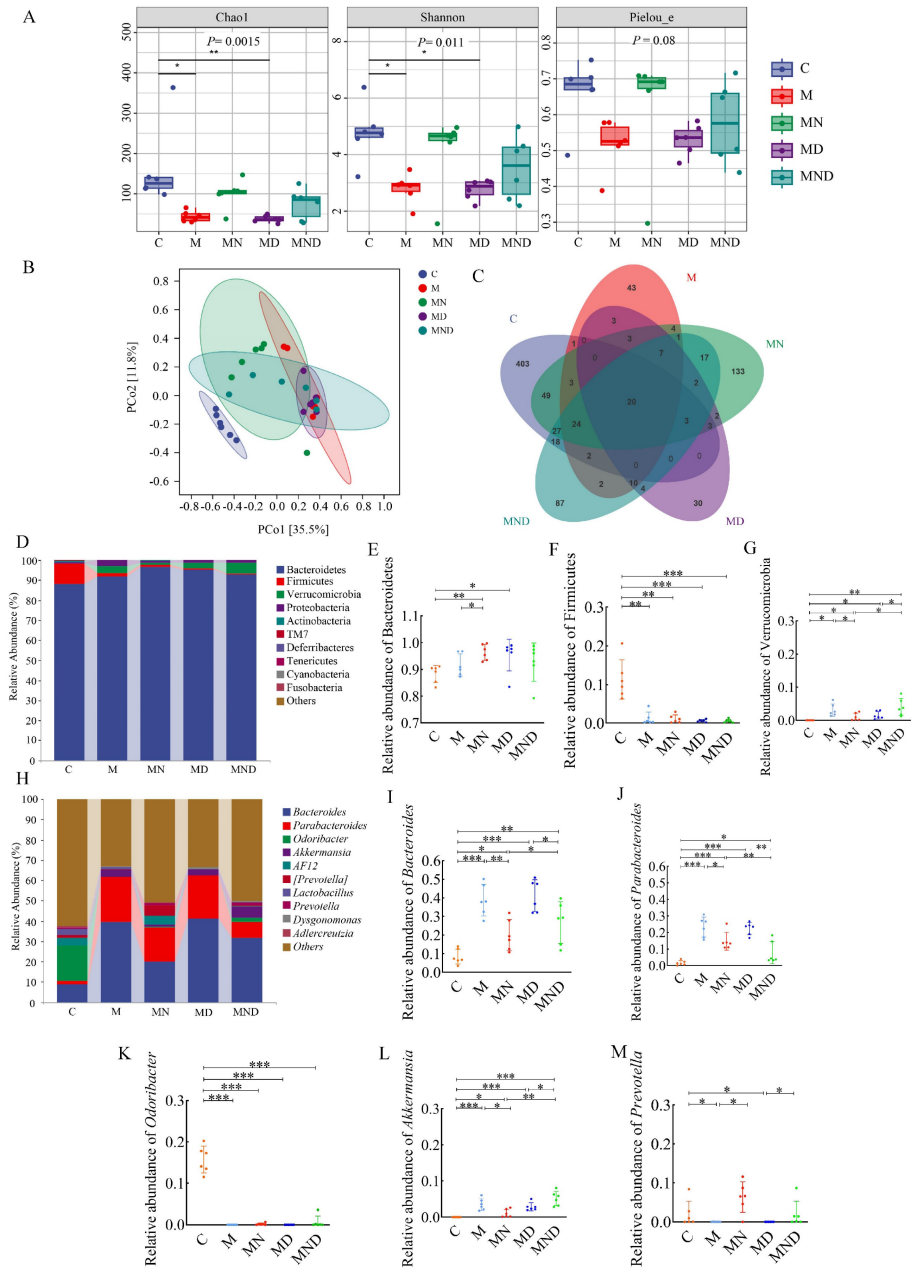
Composition of intestinal microbiome in mice treated with different drugs. (A) Chao1, Shannon and Pielou_e indexes of the alpha diversity. (A) Chao1、Shannon and Pielou_e indexes of the alpha diversity. (B) PCoA of the β-diversity index. (C) Venn map of OTUs. (D) Microbiota composition at the phylum level. The relative abundance of Bacteroidetes (E), Firmicutes (F) and Verrucomicrobic (G), n=6. (H) Microbiota composition at the genus level. The relative abundance of *Bacteroides* (I), *Parabacteroides* (J), *Odoribacter* (K), *Akkermansia* (L) and *Prevotella* (M), n = 6. *p<0.05, **p< 0.01, ***p< 0.001. C stands for blank model group, M stands for model control group, MN stands for model + naringin group, MD stands for model + DDP group, MND stands for model+ naringin+ DDP group.

To achieve this, we collected mouse faeces and analysed the bacterial and community composition. The Chao 1 metric indicates the variety within the community, while the Shannon metric indicates the overall species count. These metrics were then employed to evaluate the impact of naringin and DDP on both the α - and the β - diversity of the gut microbiota. According to the findings, group M had higher microbial diversity than DDP, whereas naringin and DDP+Naringin notably enhanced gut microbial diversity (Figure 3A). Additionally, when comparing the β-diversity among various groups using principal coordinates (PCoA) (Figure 3B), it was observed that the samples from the M and MD groups exhibited some level of proximity to each other. However, they were still significantly distinct from the MND group, indicating that the administration of chemotherapeutic drugs had a significant impact on β - diversity. This alteration resulted in a different intestinal microbial community in the mice compared to the normal mouse microbiota. The analysis of Venn diagrams revealed the presence of 20 shared OTUs among these five groups. Furthermore, the C, M, naringin, DDP and DDP+naringin groups contained 403, 43, 133, 30 and 87 unique OTUs, respectively (Figure 3C).

At the phylum level, Bacteroidetes and Firmicutes were the two most prevalent phyla, and the ratio of Firmicutes to Bacteroidetes was considerably greater in the MD group compared to the M group, but it was more similar to the M group level in the MND group (Figure 3D-G). Significant variations in the taxonomic composition of OC mouse faeces were observed at the genus level, particularly in comparison to group C (Figure 3H). Groups M, MN, MD and MND exhibited notably higher levels of *Bacteroides* and *Parabacteroides* compared to group C, with groups M and MD showing even higher levels than group MND (Figure 3I-J). Conversely, *Odoribacter* which had a higher relative abundance in group C, demonstrated significantly lower levels in the remaining four groups (Fig. 3K). Additionally, *Akkermansia* and *Prevotella* displayed a lower relative abundance in all groups, despite group C having lower levels (Figure 3L-M).

### *Bifidobacterium animalis* ameliorates tumor growth and pathological damage in mice

**Figure 4:**
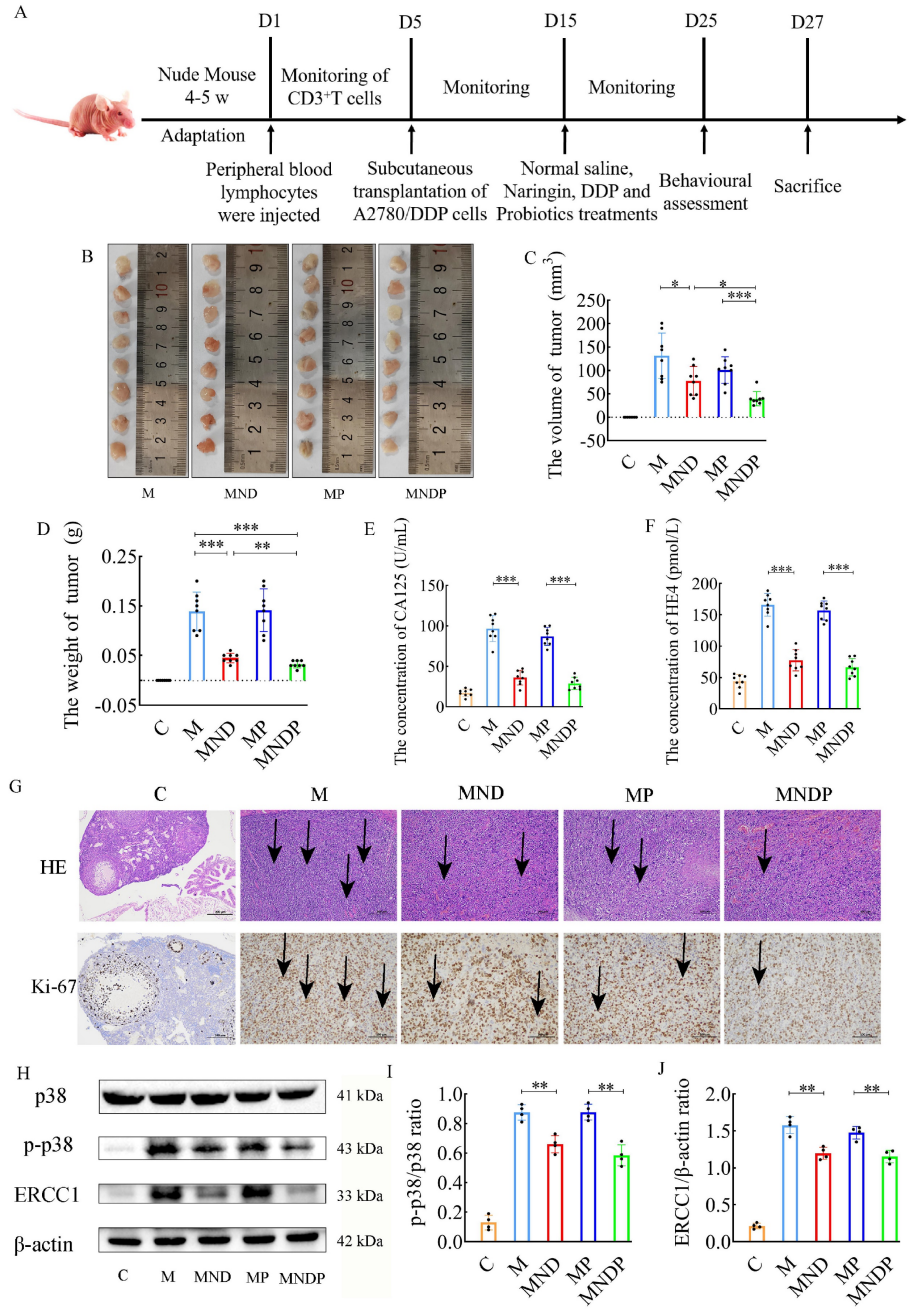
Reversal effect of combination of microbiota and naringin on cisplatin resistance in ovarian cancer in mice. (A) Schematic of animal experiments: Group C: 200 μL of sterile saline by gavage for 10 days; Group M: OC model was intragastric with 200 μL sterile saline for 10 days; Group MND: OC model was intraperitoneally injected with 200 μL cisplatin (2.5 mg/kg/d) and 200 μL naringin (2.0 mg/kg/d) for 10 days; Group MP: 10^9^ colony forming units (CFU) of *Bifidobacterium animalis* subsp. *lactis* NCU-01 continuous gavage for 10 days; Group MNDP: OC mice were intraperitoneally injected with 200 μL cisplatin (2.5 mg/kg/d) and 200 μL naringin (2.0 mg/kg/d), and 10^9^ colony forming units (CFU) of *Bifidobacterium animalis* subsp. *lactis* NCU-01 were intragastric for 10 days. (B-D) Weights and volumes of tumours in different treatment groups (n = 8). (E-F) CA125 and HE4 level in serum (n = 8). (G) H&E staining images of tumour tissues were presented (scale bar = 200 μm) and Immunohistochemistry of Ki67 expression in tumour tissues (scale bar = 100 μm). (H-J) Relative expression of p-p38/p38 and ERCC1 in tumour tissues by ImageJ software (n = 4). Data are presented as means ± SD. Student t test for two groups comparison. *p< 0.05, **p<0.01, ***p< 0.001. C stands for blank model group, M stands for model control group, MND stands for model+ naringin+ DDP group, MP stands for model + probiotic group, MNDP stands for model+ naringin+ DDP+ probiotic group.

To assess the impact of the microbiota on cancer, we performed an additional experiment using a humanized ovarian cancer model in nude mice. The complete experiment is depicted in Figure 4A. Tumor sizes and weights are shown in Figure 4B-D. *Bifidobacterium animalis* subsp. *lactis* NCU-01 alleviated tumor growth and significantly reduced tumor size compared to the other two groups using chemotherapeutic drugs and the microbiota alone. Serum CA125 and HE4 levels were decreased in the combined DDP, Naringin and NCU-01 group (MNDP) compared to the DDP and Naringin alone group (MND), whereas there was no significant change in serum CA125 and HE4 levels in the probiotic alone group (MP) compared to the M group (Figure 4E-F). Tumor histopathological H&E staining and immunohistochemical expression of Ki67 also indicated that the combined use of *Bifidobacterium animalis* subsp. *lactis* NCU-01 better inhibited the proliferation of tumor cells (Figure 4G). Moreover, chemotherapeutic drugs in combination with *Bifidobacterium animalis* subsp. *lactis* NCU-01 reduced the p-p38/p38 ratio and ERCC1 expression levels compared to those in combination with chemotherapeutic drugs only (Figure 4H-J). These results suggest that *Bifidobacterium animalis* subsp. *lactis* NCU-01 also has a certain inhibitory effect on tumor growth on the basis of conventional chemotherapeutic drug treatment and can alleviate the resistance of ovarian cancer to cisplatin and thus improve the therapeutic effect.

### *Bifidobacterium animalis* alleviates chemotherapy-induced intestinal inflammation and inhibits gastrointestinal toxicity

**Figure 5:**
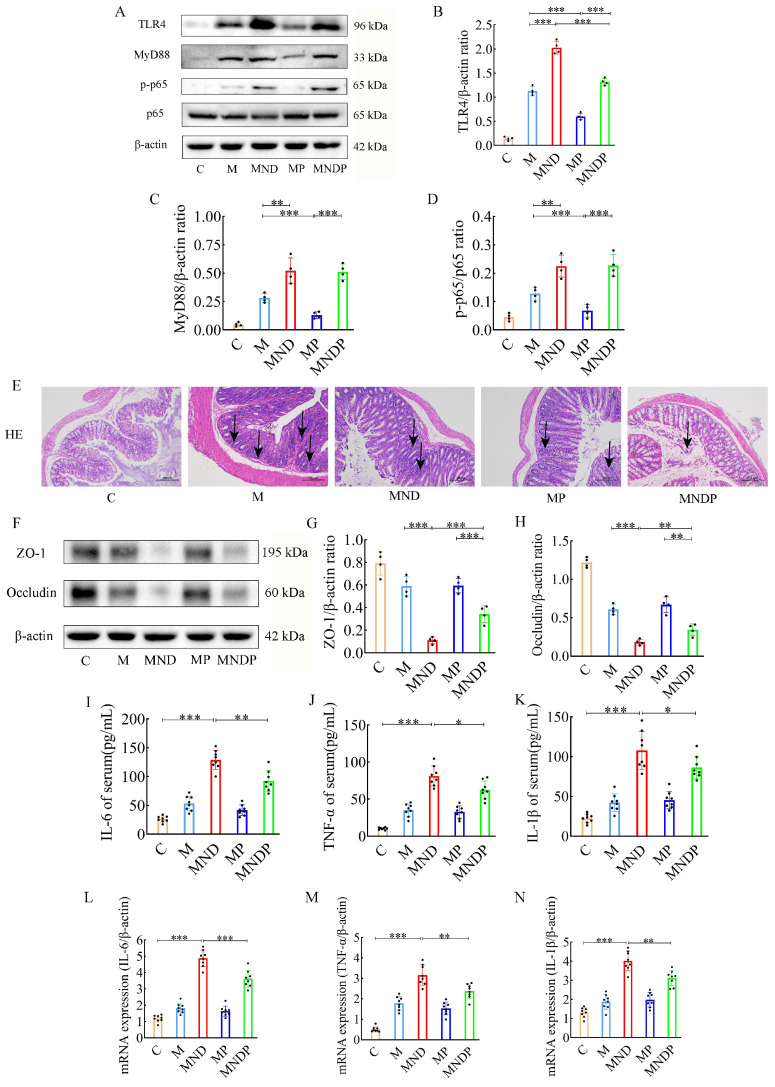
Effects of different drugs on colon of mice. (A) Effect of different drugs on the protein expression in colon tissues. (B-D) Relative expression of TLR4, MyD88 and p-p65/p65 in colonic tissues by ImageJ software (n = 4). (E) Representative H&E staining image in colon tissues (scale bar = 200 μm). (F) Effect of different drugs on the protein expression in colon tissues. (G,H) Relative expression of occludin and ZO-1 in colon tissues (n = 4). (I-N) The relative expression of IL-6, TNF-α and IL-1β detected in serum of different drugs (n = 8). Data are presented as means ± SD. Student t test for two groups comparison. *p< 0.05, **p< 0.01, ***p< 0.001. C stands for blank model group, M stands for model control group, MND stands for model+ naringin+ DDP group, MP stands for model + probiotic group, MNDP stands for model+ naringin+ DDP+ probiotic group.

Earlier research indicated that activation of the nuclear factor κB (NF-κB) and tumor necrosis factor α (TNF-α) signaling pathways are linked to intestinal injury caused by cisplatin 27. In an attempt to determine whether *Bifidobacterium animalis* subsp. *lactis* NCU-01 and naringin could attenuate DDP chemotherapy-induced gastrointestinal toxicities, we investigated pathological changes in colon tissue, inflammation-associated proteins, tight junction proteins and serum expression of inflammatory factors. Initially, we identified crucial proteins implicated in the TLR4/NF-κB inflammatory pathway, encompassing TLR4, MyD88, NF-κB (p65) and phosphorylated NF-κB (Figure 5A), using western blotting on colon tissues. The MND group exhibited elevated levels of TLR4 protein (2.030±0.132 vs. 1.127±0.089, p<0.001), MyD88 protein (1.890±0.152 vs. 1.460±0.104, *p*<0.01), and the p-p65/p65 protein ratio (0.965±0.055 vs. 0.752± 0.063, *p*<0.01), compared to the M group. Meanwhile, TLR4 protein (*p*<0.001) expression was significantly lower in the MNDP group compared to the MND group, although the MyD88 protein and p-p65/p65 protein ratios were not statistically significant to some extent (Figure 5B-D).

Histological examination using H&E staining revealed that the colonic mucosal epithelial surface in the MND group exhibited signs of damage, with a decrease in glandular structures and a notable presence of inflammatory cells infiltrating both the mucosa and the submucosa. By comparison, the M group showed less severe histopathological damage. However, the MNDP group displayed significantly amelioration of this damage (Figure 5E).

Afterwards, we measured the expression levels of ZO-1 and occludin protein, which play a crucial role in preserving tight junction integrity and barrier function, in colonic tissues (Figure 5F). In the MND group, the levels of ZO-1 protein (0.110±0.029 vs 0.590±0.086, *p*<0.001) and occludin protein (0.182±0.037 vs 0.607±0.063, *p*<0.001) were notably reduced compared to the M group. Conversely, no significant alterations were observed in the MP group. Notably, the combined use of *Bifidobacterium animalis* subsp. *lactis* NCU-01 (MNDP) restored the abundance of ZO-1 (0.342±0.076 vs 0.110±0.029, *p*<0.001) and occludin proteins when compared to the chemotherapeutic drug alone (MND) group (0.342±0.076 vs 0.182±0.037, *p*<0.01) (Figure 5G-H). To investigate the impact of the microbiota on tumor tissues and intestinal inflammation, we analysed the levels of inflammatory factors in serum. Compared with group C, in the MND group, the levels of IL-6 (129.075±16.662 vs 26.137±5.281, *p*<0.001), TNF-α (81.200±13.492 vs. 10.237±1.571, *p*<0.001), and IL-1β (107.950±23.918 vs. 22.387±5.464, *p*<0.001) were significantly higher. Similarly, the group treated with a combination of chemotherapeutic agents and NCU-01 (MNDP) showed significantly lower levels of IL-6 (*p*<0.01), TNF-α (*p*<0.05) and IL-1β (*p*<0.05) compared to the group receiving only the chemotherapeutic drug (MND) (Figure 5I-N). The above results suggest that chemotherapeutic agents cause intestinal inflammation and damage the biological barrier, inflammation through the blood circulation limits the effect of chemotherapy, and *Bifidobacterium animalis* subsp. *lactis* NCU-01 helps to alleviate inflammation, maintain the intestinal biological barrier and improve the therapeutic efficacy.

### *Bifidobacterium animalis* effectively alters the composition of intestinal microorganisms

**Figure 6:**
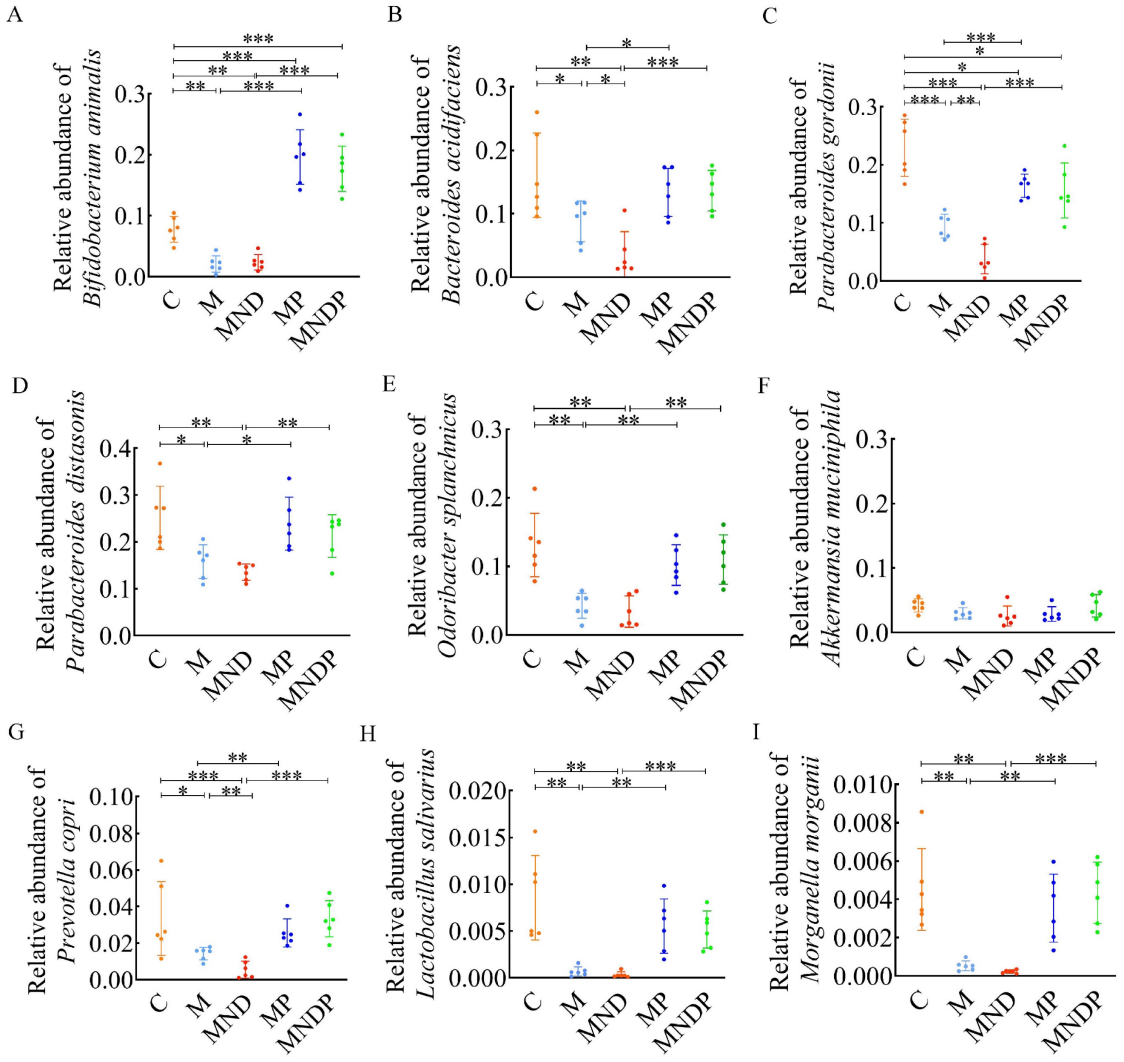
q-PCR to detect the composition of the microbiota in the gut of mice after intervention. (A) Abundance of *Bifidobacterium animals*; (B) Abundance of *Bacteroides acidifaciens*; (C) Abundance of *Parabacteroides gordonii*; (D) Abundance of *Parabacteroides distasonis*; (E) Abundance of *Odoribacter splanchnicus*; (F) Abundance of *Akkermansia muciniphila*; (G) Abundance of *Prevotella copri*; (H) Abundance of *Lactobacillus salivarius*; (I) Abundance of *Morganella morganii*.

To determine the impact of *Bifidobacterium animalis* subsp. *lactis* NCU-01 on the disruption of intestinal microecology in mice, we gathered stool samples and conducted real-time quantitative PCR examination to identify alterations in gut microorganisms. The findings indicated that the proportion of *Bifidobacteria* in the MP and MNDP groups was considerably greater than that in the M and MND groups. However, the proportion of *Bifidobacteria* in the MP group surpassed that in the MNDP group (Figure 6A). In comparison to the M and MND groups, there was a substantial increase in the proportion of *Bacteroides acidifaciens*, *Parabacteroides gordonii*, *Parabacteroides distasonis*, and *Odoribacter splanchnicus* in group MP and MNDP. Additionally, the proportions in the MP and MNDP groups were similar to that of group C (Figure 6B-E). Despite the lack of a statistically significant distinction in *Akkermansia mucinphila* levels among the five groups, the MNDP group exhibited a higher relative abundance in comparison to the MND group (Figure 6F). Furthermore, *Prevotella copri*, *Lactobacillus salivarius* and *Morganella morganii*, which exhibited higher relative abundances in group C, experienced relative increases in both the MP and MNDP groups, surpassing the levels observed in groups M and MND (Figure 6G-I). The above data suggest that *Bifidobacterium animalis* subsp. *lactis* NCU-01 has the ability to enhance the quantity of advantageous microorganisms, ameliorate the imbalance in the intestinal microecology and alleviate inflammation in the intestines.

## Discussion

Cisplatin can inhibit cancer cells that cannot be removed surgically or have spread far away, which has a crucial role in improving patient prognosis. However, despite its effectiveness, approximately 80% of patients experience relapse after chemotherapy due to cisplatin resistance 28,29. Furthermore, cisplatin and its byproducts cause severe harm to the intestinal mucosa, leading to various gastrointestinal side effects in nearly all patients, thereby posing significant risks to patient well-being 2,30. In the field of cancer therapy, combining herbal medicine with chemotherapy may have important advantages. Chinese medicines can enhance the toxic effects of chemotherapeutic drugs against tumors implanted in vivo as well as OC cells cultured in vitro, reduce the resistance of OC cells to chemotherapy by acting on multiple biological targets, and restore and promote programmed death of cancer cells, thus effectively inhibiting tumor growth. Hence, this study delved into a novel treatment approach aimed at reducing drug resistance and the adverse effects on the digestive system caused by cisplatin, ultimately leading to an enhanced patient survival rate.

In our prior investigation, it was shown that naringin effectively suppressed growth and enhanced the responsiveness to cisplatin treatment in SKOV3/cDDP cells, which had developed resistance to cisplatin 31. However, the precise mechanism through which naringin overcomes cisplatin resistance is still unknown. In order to further authenticate and explore the functioning of naringin on OC, we conducted *in vitro* tests to verify that naringin had the ability to enhance the responsiveness of the A2780/DDP ovarian cancer cell line to cisplatin. Additionally, we found that this impact relied on the activation of MAPK (Figure 1). In comparison to DDP alone, the combination of naringin and DDP effectively reversed the resistance of ovarian cancer cells to cisplatin. This combination also presented initial proof suggesting that naringin could function as a p38 inhibitor, hindering the expression of ERCC1 by obstructing the p38 signaling pathway. Consequently, this inhibition enhances the sensitivity of ovarian cancer to cisplatin. ERCC1, an essential enzyme involved in DNA repair, acts as a single-stranded DNA endonuclease. The chemotherapeutic response is significantly influenced by the pivotal involvement of p38MAPK, as discovered by researchers. The involvement of this route has been linked to the programmed cell death of cancer cells and is triggered by various chemotherapy drugs 32. Blocking p38MAPK has been suggested as a strategy to identify resistance to cisplatin in multiple experimental models 33,34.

To address the issue of human tumor studies lacking accurate representation of the human immune system, we utilized our knowledge from creating the HuPBL-SCID murine chimera model 35 to develop a humanized nude mouse model. This involved intraperitoneal injection of human peripheral blood T-lymphocytes (HuPBTL) into nude mice, aiming to restore their cellular immunity. The resulting model was named the HuPBTL-nude mouse chimera. To determine if human cellular immunity has been successfully restored in nude mice, the FCM assay can be used to detect specific markers for humanized T cells such as CD3^+^ T cells, CD4^+^ T cells and CD8^+^ T cells in the peripheral blood or lymphoid organs (e.g. spleen) of the mice 35. The study found that human CD3^+^ T cells were present in the peripheral blood of nude mice in the experimental group (Figure 2B-I). After immune reconstitution, the tumorigenicity rate was high and had no significant impact on tumor growth. This nude mouse model, called Human Ovarian Carcinoma-HuPBTL-nude mice, successfully simulated tumors in patients with ovarian cancer and certain cellular immune function. It accurately reflects the characteristics of the human immune system, which previous animal models failed to do. Additionally, compared to HuPBL-SCID mice, nude mice have better feeding conditions, lower infection and mortality rates, and reduced experimental costs. To summarize, the HuPBTL-nude mouse model of human ovarian cancer can replicate the tumor condition seen in ovarian cancer patients who possess specific cellular immune capabilities. This model serves as an excellent tool for investigating ovarian cancer treatment, enabling objective and precise assessment of therapeutic outcomes. Additionally, it offers a fresh perspective and approach to developing alternative animal models for tumor treatment.

Building upon prior experiments, using human ovarian cancer cells, we created an OC mouse model to examine the impact and mechanism of naringin in reducing resistance to DDP. In previous studies, we used intraperitoneal injection of naringin to treat subcutaneous transplanted tumors in nude mice. Although chemotherapy drugs are administered intravenously in clinical work, due to the difficulty of intravenous administration in nude mice, we still chose intraperitoneal injection of naringin to observe the growth of subcutaneous transplanted tumors in humanized nude mice. The findings indicated that the pairing of naringin and cisplatin effectively decreased the dimensions of transplanted tumors, the levels of tumor markers CA125 and HE4, along with the heterogeneity of cancerous cells and the proportion of Ki67-positive cells in tissue samples. In the MND group, protein expression of p-p38MAPK and ERCC1 was significantly decreased, similar to the findings from *in vitro* cellular experiments (Figure 2J-Q). The glycoprotein CA125 is frequently employed for the diagnosis and tracking of epithelial ovarian cancer. HE4, an acidic whey protein, may be increased in various cancers. Studies have indicated that evaluating CA125 and HE4 together yields more precise outcomes for early OC detection compared to individual tests 36,37. Consequently, it is crucial to simultaneously detect levels of CA125 and HE4 to enhance survival rates and minimize treatment expenses. We previously conducted cellular experiments *in vitro* to examine the activation of the p38MAPK signaling pathway, as depicted in figure 1, and obtained comparable outcomes in mice with ovarian cancer. Therefore, we conclude that naringin is able to eliminate cisplatin resistance in ovarian cancer by modulating the p38 signaling pathway and decreasing ERCC1 expression in cells.

In recent years, there has been a correlation between the gut microbiota and the development and treatment of cancer 23-26,38,39. In order to gain a deeper comprehension of the impacts of naringin and DDP on the gut microbiota, we conducted a comparison of the diversity of gut microbiota composition among regular mice, OC mice and mice that received a combination of naringin and cisplatin treatment, utilizing high-throughput sequencing of 16S rRNA. Based on our findings, OC mice exhibited dysbiosis, characterized by reduced diversity, an elevated presence of opportunistic pathogens, and a lower abundance of beneficial bacteria (Figure 3). In comparison to the M group, the MND group exhibited a higher abundance of Bacteroidetes (particularly *Bacteroides* and *Prevotella*), while showing a lower abundance of Firmicutes. In a previous investigation, the ratios of Proteobacteria to Firmicutes were notably elevated in OC tissues compared to normal oviduct samples 40. Patients with gastrointestinal cancers exhibited comparable alterations in their gut microbiota, such as a rise in *Bacteroides* and *Akkermansia genera*, alongside a decline in *Bifidobacterium* 41. *Bifidobacterium* is an important producer of butyrate, which is known to have strong anti-inflammatory and anti-cancer effects, and increasing butyrate levels in the body has been shown to significantly reduce the risk of tumors triggered by carcinogens 42.

*Bifidobacteria*, a crucial member of the microbiota group, possess the ability to hinder pathogens, modulate the immune system, and uphold the balance of the intestinal microbiota 43. Previous studies have demonstrated that oral intake of *Bifidobacteria* can alleviate gastrointestinal symptoms, lessen inflammatory reactions and facilitate the recovery of gut microbiota diversity in postoperative cancer patients 44. However, there is currently no evidence supporting the involvement of *Bifidobacteria* in the recuperation and response to gastrointestinal toxicity in ovarian cancer patients undergoing cisplatin chemotherapy, we hypothesize that *Bifidobacteria* could potentially play a crucial role in enhancing the anticancer properties of naringin. To test this hypothesis, we investigated the potential therapeutic mechanisms and primary function of *Bifidobacteria* in a humanized nude mouse model of ovarian cancer. In comparison to the MND group, the MNDP group exhibited the lowest tumor volume, a more significant decrease in the levels of the tumor markers CA125 and HE4, a further reduction in cellular isoforms and the percentage of Ki67-positive cells and a greater decrease in the expression of the p38 pathway-related proteins p-p38 and ERCC1 (Figure 4). *Bifidobacterium animalis* subsp. *lactis* NCU-01 could potentially play a crucial role in overcoming cisplatin resistance in naringin.

In Figure 5 *Bifidobacterium animalis* subsp. *lactis* NCU-01 reduced the levels of inflammatory proteins TLR4, MyD88 and p-p65 while enhancing the expression of tight junction proteins ZO-1 and occludin. Furthermore, it led to a decrease in the proportion of inflammatory cells in the tissue sections. Additionally, we employed an ELISA kit to quantify the serum levels of pro-inflammatory cytokines IL-6, IL-1β and TNF-α. According to the findings, the addition of microbiota notably decreased the levels of IL-6, IL-1β, and TNF-α in comparison to the MND group. Radiation and chemotherapy have been found to cause harm to blood vessels, epithelial cells, and tissues in numerous research studies 45. Furthermore, they promote the production of reactive oxygen species, which then activate the TLR4/NF-κB pathway, boost the production of inflammatory substances (TNF-α, IL-1β and IL-6), accelerate apoptosis (Bax/Bcl-2) and ultimately result in heightened tissue harm and susceptibility to bacterial, viral and fungal infections 46,47.

In further studies, we analysed the intestinal microbiota in the faeces of these groups of mice. In healthy adults, *Bifidobacterium* is a major intestinal commensal that reduces inflammation and suppresses tumors after intestinal damage 44. In comparison to MND, MNDP exhibited higher levels of advantageous microorganisms such as *Bifidobacterium animalis*, *Parabacteroides distasonis* and *Prevotella copri*. Although the alterations in *Akkermansia muciniphila* were not statistically significant, all of these microorganisms displayed an increase (Figure 6). Previous studies have shown that *Bacteroides* are linked to mucolytic and pro-inflammatory characteristics 48. Furthermore, several studies have indicated that *Akkermansia muciniphila* could potentially serve as a beneficial microbe for the intestinal mucosa 49. Conversely, a scarcity of *Akkermansia muciniphila* might compromise the integrity of the intestinal mucosa, enabling the infiltration of harmful elements such as lipopolysaccharides (LPS) across the epithelial barrier. However, it should be noted that this hypothesis has not been verified as of yet.

## Conclusions

**Figure 7:**
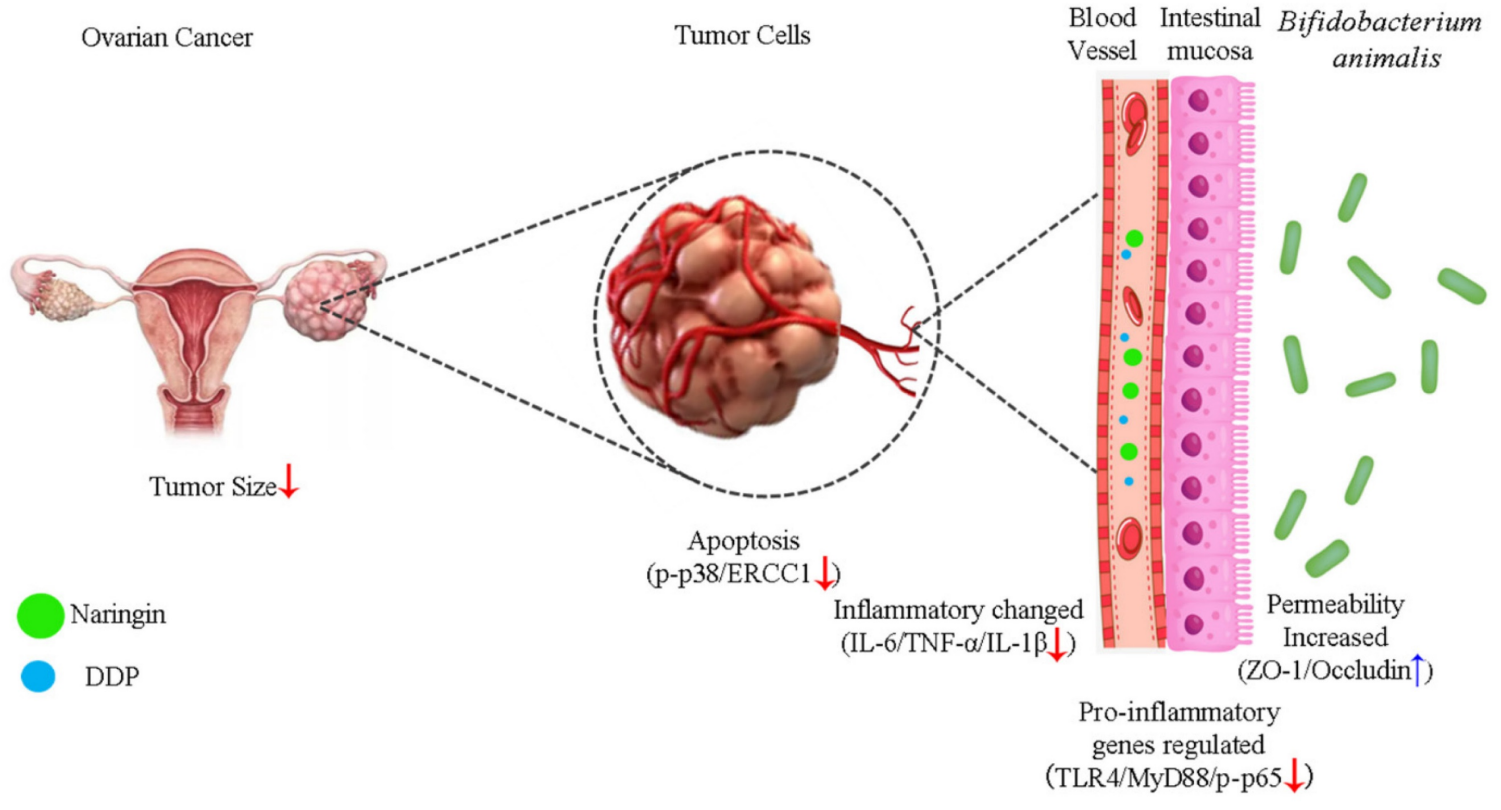
Schematic illustration of the potential mechanism of naringin combined with *Bifidobacterium animalis* subsp. *lactis* NCU-01 to reverse cisplatin resistance in ovarian cancer. NCU-01 can increase the expression of intestinal permeability-related proteins ZO-1 and occludin by regulate intestinal microbiota, inhibit TLR4/NF-κB-induced intestinal inflammation, and then inhibit the p38MAPK signaling pathway through blood circulation, downregulate the expression of ERCC1, and reverse cisplatin resistance in ovarian cancer.

Our results showed that naringin and NCU-01, combined with DDP, could regulate the TLR4/NF-κB/p38MAPK signaling pathway, repair the damage causing increase intestinal permeability and regulate the composition of intestinal probiotic, which effectively reversed the resistance of ovarian cancer to platinum-based chemotherapeutic agents (Figure 7), and provided a possible drug for the clinical treatment of OC and the reduction of the associated gastrointestinal side effects. However, additional studies are needed to explore the role of gut microbiota dysbiosis in systemic inflammation, as well as to investigate the precise mechanisms of the interaction between the NF-κB and p38MAPK signaling pathways. In addition, further studies are needed to examine whether other factors influence the composition of the gut microbiota and thus the susceptibility of ovarian cancer to platinum-based chemotherapeutic agents.

## Supplementary Material

Supplementary tables.

## References

[1] Ma Y, Zheng W (2021). H3K27ac-induced incRNA PAXIP1-AS1 promotes cell proliferation, migration, EMT and apoptosis in ovarian cancer by targeting miR-6744-5p/PCBP2 axis. J Ovarian Res.

[2] Ghosh S (2019). Cisplatin: The first metal based anticancer drug. Bioorg Chem.

[3] Pujade-Lauraine E, Hilpert F, Weber B (2014). Bevacizumab combined with chemotherapy for platinum-resistant recurrent ovarian cancer: The AURELIA open-label randomized phase III trial. J Clin Oncol.

[4] Pujade-Lauraine E, J Alexandre (2011). Update of randomized trials in recurrent disease. Ann Oncol.

[5] Ahmad N, Qamar M, Yuan Y (2022). Dietary polyphenols: extraction, identification, bioavailability, and role for prevention and treatment of colorectal and prostate cancers. Molecules.

[6] Khan UM, Sameen A, Aadil RM (2021). Citrus genus and its waste utilization: A review on Health-promoting activities and industrial application. Evid Based Complement Alternat Med.

[7] Alam F, Mohammadin K, Shafique Z (2022). Citrus flavonoids as potential therapeutic agents: A review. Phytother Res.

[8] Cirmi S, Ferlazzo N, Lombardo GE (2016). Chemopreventive agents and inhibitors of cancer hallmarks: May citrus offer new perspectives?. Nutrients.

[9] Bharti S, Rani N, Krishnamurthy B (2014). Preclinical evidence for the pharmacological actions of naringin: A review. Planta Med.

[10] Ghanbari-Movahed M, Jackson G, Farzaei MH (2021). A systematic review of the preventive and therapeutic effects of naringin against human malignancies. Front Pharmacol.

[11] de Vos WM, Tilg H, Van Hul M (2022). Gut microbiome and health: Mechanistic insights. Gut.

[12] Zhou X, Lu J, Wei K (2021). Neuroprotective effect of ceftriaxone on MPTP-induced Parkinson's disease mouse model by regulating inflammation and intestinal microbiota. Oxid Med Cell Longev.

[13] Wang Xu-Wen, Yang-Yu Liu (2020). Comparative study of classifiers for human microbiome data. Med Microecol.

[14] Lakritz JR, Poutahidis T, Levkovich T (2014). Beneficial bacteria stimulate host immune cells to counteract dietary and genetic predisposition to mammary cancer in mice. Int J Cancer.

[15] Vétizou M, Pitt JM, Daillère R (2015). Anticancer immunotherapy by CTLA-4 blockade relies on the gut microbiota. Science.

[16] Daillère R, Vétizou M, Waldschmitt N (2016). Enterococcus hirae and barnesiella intestinihominis facilitate cyclophosphamide-induced therapeutic immunomodulatory effects. Immunity.

[17] He BL, Xiong Y, Hu TG (2023). *Bifidobacterium spp*. as functional foods: A review of current status, challenges, and strategies. Crit Rev Food Sci Nutr.

[18] Chung L, Thiele Orberg E, Geis AL (2018). *Bacteroides* fragilis toxin coordinates a pro-carcinogenic inflammatory cascade via targeting of colonic epithelial cells. Cell Host Microbe.

[19] Jungersen M, Wind A, Johansen E (2014). The science behind the probiotic strain *bifidobacterium animalis* subsp. *lactis* BB-12(®). Microorganisms.

[20] Fan B, Wang YY, Zhao YX (2022). Discussion on the construction technology of mouse model with mumanized immune. Curr Biotechnol.

[21] Rios-Doria J, Stevens C, Maddage C (2020). Characterization of human cancer xenografts in humanized mice. J Immunother Cancer.

[22] Greenfield EA (2019). Administering anesthesia to mice, rats, and hamsters. Cold Spring Harb Protoc.

[23] Gopalakrishnan V, Helmink BA, Spencer CN (2018). The influence of the gut microbiome on cancer, immunity, and cancer immunotherapy. Cancer Cell.

[24] Goubet AG, Daillère R, Routy B (2018). The impact of the intestinal microbiota in therapeutic responses against cancer. C R Biol.

[25] Iida N, Dzutsev A, Stewart CA (2013). Commensal bacteria control cancer response to therapy by modulating the tumor microenvironment. Science.

[26] Routy B, Le Chatelier E, Derosa L (2018). Gut microbiome influences efficacy of PD-1-based immunotherapy against epithelial tumors. Science.

[27] Lee CS, Ryan EJ, Doherty GA (2014). Gastro-intestinal toxicity of chemotherapeutics in colorectal cancer: the role of inflammation. World J Gastroenterol.

[28] Alexandrova E, Pecoraro G, Sellitto A (2020). An overview of candidate therapeutic target genes in ovarian cancer. Cancers (Basel).

[29] Stewart C, Ralyea C, Lockwood S (2019). Ovarian cancer: an integrated review. Semin Oncol Nurs.

[30] Hu JN, Yang JY, Jiang S (2021). Panax quinquefolium saponins protect against cisplatin evoked intestinal injury via ROS-mediated multiple mechanisms. Phytomedicine.

[31] Zhu H, Zou X, Lin S (2020). Effects of naringin on reversing cisplatin resistance and the Wnt/β-catenin pathway in human ovarian cancer SKOV3/CDDP cells. J Int Med Res.

[32] Zhang B, Wang X, Cai F (2013). Antitumor properties of salinomycin on cisplatin-resistant human ovarian cancer cells *in vitro* and *in vivo*: involvement of p38 MAPK activation. Oncol Rep.

[33] Cuadrado A, Lafarga V, Cheung PC (2007). A new p38 MAP kinase-regulated transcriptional coactivator that stimulates p53-dependent apoptosis. EMBO J.

[34] St Germain C, Niknejad N, Ma L (2010). Cisplatin induces cytotoxicity through the mitogen-activated protein kinase pathways and activating transcription factor 3. Neoplasia.

[35] Matsuyama M, Yoshimura R, Mitsuhashi M (2005). 5-Lipoxygenase inhibitors attenuate growth of human renal cell carcinoma and induce apoptosis through arachidonic acid pathway. Oncol Rep.

[36] Moore RG, Brown AK, Miller MC (2008). The use of multiple novel tumor biomarkers for the detection of ovarian carcinoma in patients with a pelvic mass. Gynecol Oncol.

[37] Moore RG, McMeekin DS, Brown AK (2009). A novel multiple marker bioassay utilizing HE4 and CA125 for the prediction of ovarian cancer in patients with a pelvic mass. Gynecol Oncol.

[38] Gopalakrishnan V, Spencer CN, Nezi L (2018). Gut microbiome modulates response to anti-PD-1 immunotherapy in melanoma patients. Science.

[39] Kuczma MP, Ding ZC, Li T (2017). The impact of antibiotic usage on the efficacy of chemoimmunotherapy is contingent on the source of tumor-reactive T cells. Oncotarget.

[40] Zhou B, Sun C, Huang J (2019). The biodiversity composition of microbiome in ovarian carcinoma patients. Sci Rep.

[41] Li N, Bai C, Zhao L (2021). The relationship between gut microbiome features and chemotherapy response in gastrointestinal cancer. Front Oncol.

[42] Bozkurt HS, Quigley EM, Kara B (2019). *Bifidobacterium animalis* subspecies *lactis* engineered to produce mycosporin-like amino acids in colorectal cancer prevention. SAGE Open Med.

[43] Alessandri G, van Sinderen D, Ventura M (2021). The genus *bifidobacterium*: from genomics to functionality of an important component of the mammalian gut microbiota running title: *Bifidobacterial* adaptation to and interaction with the host. Comput Struct Biotechnol J.

[44] Zheng C, Chen T, Wang Y (2019). A randomised trial of probiotics to reduce severity of physiological and microbial disorders induced by partial gastrectomy for patients with gastric cancer. J Cancer.

[45] Clemente AM, Rizzetto L, Castronovo G (2015). Effects of near-infrared laser radiation on the survival and inflammatory potential of *Candida spp*. involved in the pathogenesis of chemotherapy-induced oral mucositis. Eur J Clin Microbiol Infect Dis.

[46] Tancharoen S, Shakya P, Narkpinit S (2018). Anthocyanins extracted from oryza sativa L. Prevent fluorouracil-induced nuclear factor-κB activation in oral mucositis: *In vitro* and *in vivo* studies. Int J Mol Sci.

[47] Luo J, Bian L, Blevins MA (2019). Smad7 promotes healing of radiotherapy-induced oral mucositis without compromising oral cancer therapy in a xenograft mouse model. Clin Cancer Res.

[48] Le Chatelier E, Nielsen T, Qin J (2013). MetaHIT consortium: Richness of human gut microbiome correlates with metabolic markers. Nature.

[49] Bedarf JR, Hildebrand F, Coelho LP (2017). Functional implications of microbial and viral gut metagenome changes in early stage L-DOPA-naïve Parkinson's disease patients. Genome Med.

