# Supplementary material for: Reversal of Platinum-based Chemotherapy Resistance in Ovarian Cancer by Naringin Through Modulation of The Gut Microbiota in a Humanized Nude Mouse Model
Source: J Cancer. 2024 Jun 11;15(13):4430–47. doi: 10.7150/jca.96448 (PMC11212103; doi:10.7150/jca.96448)
Supplement: Supplementary file 1 — Supplementary tables. [file jcav15p4430s1.pdf]

Table SI. Antibodies used in this study

Antibodies	Source/Cat.No	Host	Dilution
P38 Mitogen-activated protein kinase (P38 MAPK)	Abmart(T55600F)	Rabbit	1:2,000(WB)
Phospho-P38 Mitogen-activated protein kinase (p-P38 MAPK)	Abmart(TP56391F)	Rabbit	1:2,000(WB)
Excision repair cross complementing group 1 (ERCC1)	Proteintech(14586-1-AP)	Rabbit	1:2,500(WB)
Ki-67	Servicebio(GB111141)	Rabbit	1:500(IHC)
β-actin	CST(4970S)	Rabbit	1:1,000(WB)
Nuclear factor-ĸB p65 (NF-ĸB p65)	Proteintech(66535-1-Ig)	Mouse	1:1,000(WB)
Phospho-Nuclear factor-ĸB p65 (p-NF-ĸB p65)	CST(3033S)	Rabbit	1:1,000(WB)
Myeloid differentiation factor 88 (MYD88)	Proteintech(23230-1-AP)	Rabbit	1:1,000(WB)
Toll-like receptor 4 (TLR4)	Proteintech(66350-1-Ig)	Mouse	1:4,000(WB)
Occludin	Proteintech(13409-1-AP)	Rabbit	1:1,500(WB)
Zonula occludens-1 (ZO-1)	Abcam(ab96587)	Rabbit	1:3,000(WB)

Immunohistochemical secondary antibody: HRP-labeled anti-rabbit antibody (Servicebio, Catalog No. GB23303)

WB secondary antibody: goat anti-rabbit (Abcam, 205718) or goat anti-mouse (Abcam, 97265)

Table SII. Primer sequence used in this study

Gene name	Primer sequence
Bifidobacterium animalis-Forward primer	5 '-actgcaactgtgcttgct-3 '
Bifidobacterium animalis-Reverse primer	5 '-cttgaaggtgccacggat-3 '
Bacteroides acidifaciens-Forward primer	5 '-tagtccctgaaaccatgcgc-3 '
Bacteroides acidifaciens-Reverse primer	5 '-actaatgcgcattcggagtt-3 '
Parabacteroides gordonii-Forward primer	5 '-ataacccggcgaaagtcgga-3 '
Parabacteroides gordonii-Reverse primer	5 '-agcatcacatcgctgtgtag-3 '
Parabacteroides distasonis-Forward primer	5 '-ctcgattgcggctgcaaa-3 '
Parabacteroides distasonis-Reverse primer	5 '-agagaacggcgcttcaga-3 '
Odoribacter splanchnicus-Forward primer	5 '-aggctctcagaggcgatg-3 '
Odoribacter splanchnicus-Reverse primer	5 '-cgctcgactagtgagctg-3 '
Akkermansia mucinphila-Forward primer	5 '-tagccctgggaaactggg-3 '
Akkermansia mucinphila-Reverse primer	5 '-tcttgcgaccgtactccc-3 '
Prevotella copri-Forward primer	5 '-gcgtatccaacctgccca-3 '
Prevotella copri-Reverse primer	5 '-gtgagctgccttcgcaat-3 '
Lactobacillus salivarius-Forward primer	5 '-ctgctggtgcaaacccag-3 '
Lactobacillus salivarius-Reverse primer	5 '-gctgtaccaccaccagga-3 '
Morganella morganii-Forward primer	5 '-tgcatctctccgccctac-3 '
Morganella morganii-Reverse primer	5 '-ggctctgtccgagctgat-3 '
